# Association of C-Reactive Protein with Functional Outcome After Acute Ischemic Stroke Across TOAST Subtypes

**DOI:** 10.3390/jcm15187192

**Published:** 2026-09-16

**Authors:** Yoonji Oh, Jeong Ung Jeong, Sang-Hun Lee, Moon-Ho Park

**Affiliations:** 1 Department of Medical Science, Graduate School of Medicine, Korea University, Seoul 02841, Republic of Korea; 2 Department of Obstetrics and Gynecology, Korea University Guro Hospital, Korea University College of Medicine, Seoul 08308, Republic of Korea; 3 Department of Neurology, Korea University Ansan Hospital, Korea University College of Medicine, Ansan 15355, Republic of Korea

**Keywords:** stroke, CRP, prognosis, TOAST

## Abstract

**Background/Objectives**: Elevated C-reactive protein (CRP) has been associated with adverse outcomes after acute ischemic stroke (AIS), but whether the association differs by stroke etiology remains uncertain. We evaluated elevated CRP in relation to 3-month functional outcome across Trial of Org 10172 in Acute Stroke Treatment (TOAST) subtypes. **Methods**: This retrospective study included 2975 consecutive patients with AIS from a stroke registry. Elevated CRP was defined as ≥3 mg/L, and the primary outcome was an unfavorable 3-month modified Rankin Scale (mRS) score of 2–6. Multivariable logistic regression was performed overall and within TOAST subtypes. Sensitivity analyses used raw NIHSS, continuous CRP, alternative CRP thresholds, an alternative mRS cutoff, and formal CRP×TOAST interaction testing. **Results**: Overall, 1464 patients (49.2%) had an unfavorable outcome. In the prespecified model, elevated CRP was associated with an unfavorable outcome overall (adjusted odds ratio [aOR], 1.301; 95% confidence interval [CI], 1.003–1.687; *p* = 0.047) and in the small-vessel occlusion (SVO) subgroup (aOR, 2.620; 95% CI, 1.094–6.275; *p* = 0.031). However, the all-category CRP×TOAST interaction was not significant (P_interaction_ = 0.154). With nonlinear adjustment for raw NIHSS, the SVO association was attenuated (aOR, 2.243; 95% CI, 0.899–5.598; *p* = 0.083), and the interaction remained nonsignificant (P_interaction_ = 0.317). **Conclusions**: Higher CRP was associated with unfavorable functional outcome, but the findings did not establish a TOAST subtype-specific association.

## 1. Introduction

Systemic inflammation after acute ischemic stroke contributes substantially to secondary brain injury and has been implicated in impaired neurological recovery [1,2]. Inflammatory responses exacerbate blood–brain barrier disruption, neuronal injury, and tissue damage, leading to poorer functional outcomes [1,3]. Accordingly, elevated levels of C-reactive protein (CRP), a widely used marker of systemic inflammation, have consistently been associated with adverse outcomes after ischemic stroke [4,5,6,7].

Despite these observations, whether the prognostic significance of CRP varies according to stroke etiology remains uncertain. Acute ischemic stroke comprises several etiologically distinct subtypes, including large-artery disease (LAD), cardioembolism (CE), and small-vessel occlusion (SVO), each characterized by different mechanisms of vascular injury, thrombus formation, and tissue vulnerability [8]. Most previous studies have evaluated inflammatory biomarkers in unselected stroke populations, providing limited insight into subtype-specific associations [4,5,6]. If CRP reflects pathophysiological mechanisms that differ across stroke etiologies, its prognostic value may also vary among stroke subtypes. Clarifying these differences may improve risk stratification and help identify patients most likely to benefit from targeted preventive strategies.

In this study, we investigated the association between CRP measured from fasting blood obtained on the morning after admission and 3-month functional outcome in patients with acute ischemic stroke. We also explored the pattern of this association across TOAST subtypes and formally tested CRP×TOAST interaction.

## 2. Materials and Methods

### 2.1. Participants

**Figure 1:**
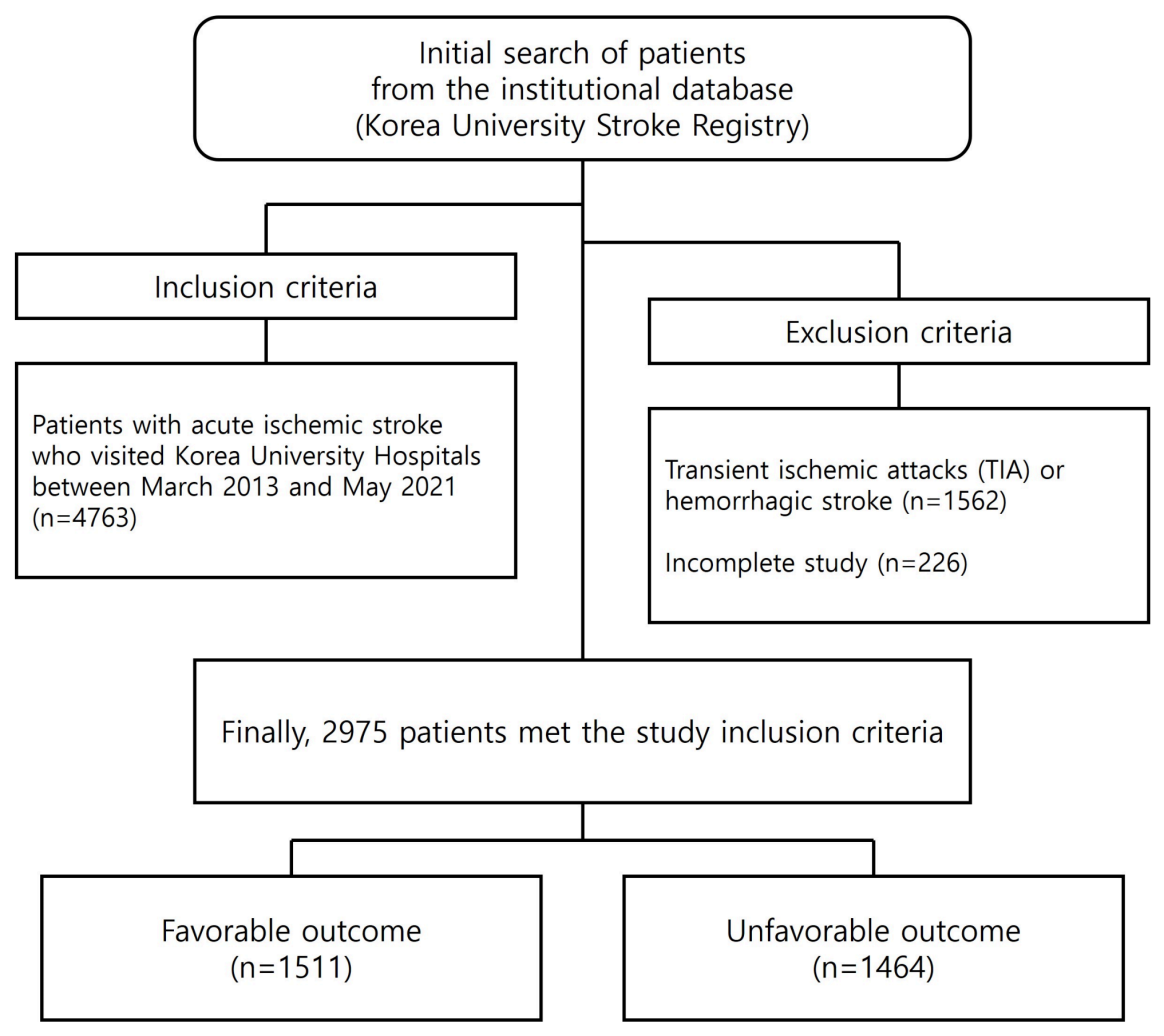
Flowchart of patient selection. Incomplete data were identified for dyslipidemia (*n* = 95), initial NIHSS score (*n* = 20), WBC count (*n* = 4), anemia (*n* = 4), eGFR (*n* = 2), and CRP (*n* = 162). Because some patients had missing data for more than one variable, these categories were not mutually exclusive, resulting in 226 unique patients excluded because of incomplete data.

This retrospective study used anonymized data from the Korea University Stroke Registry [9]. Stroke was defined, according to the World Health Organization criteria [10], as rapidly developing clinical signs of focal or global cerebral dysfunction lasting at least 24 h or leading to death without any apparent non-vascular cause. Ischemic stroke was diagnosed based on a compatible clinical history and neurological findings together with diffusion-weighted MRI, demonstrating corresponding ischemic lesions with a reduced apparent diffusion coefficient. We retrospectively included consecutive patients with acute ischemic stroke who were diagnosed by a neurologist within 30 days after symptom onset and admitted to the Stroke Center of Korea University Ansan Hospital between March 2013 and May 2021. Patients with transient ischemic attacks or hemorrhagic strokes were excluded from the study (Figure 1). 

Demographic, clinical, laboratory, neuroimaging, and outcome data were collected. Brain CT and/or MRI were performed to exclude intracranial hemorrhage and other stroke mimics, and all patients underwent at least one vascular imaging study, including conventional angiography, MR angiography, CT angiography, or carotid duplex ultrasonography. Routine evaluations included laboratory testing, chest radiography, and 12-lead electrocardiography. Additional investigations, including transcranial Doppler ultrasonography, transthoracic or transesophageal echocardiography, carotid duplex ultrasonography, and 24 h Holter monitoring, were performed when clinically indicated.

The study was approved by the Institutional Review Board of Korea University Ansan Hospital (Approval No. AS0213), which waived the requirement for informed consent because of the retrospective study design.

### 2.2. Comorbidities and Clinical Characteristics

Comorbidities were recorded using predefined criteria, including hypertension (systolic blood pressure ≥ 140 mmHg, diastolic blood pressure ≥ 90 mmHg, or use of antihypertensive medication), diabetes mellitus (fasting plasma glucose ≥ 126 mg/dL, non-fasting plasma glucose ≥ 200 mg/dL, or use of glucose-lowering medication), dyslipidemia (total cholesterol ≥ 200 mg/dL, low-density lipoprotein cholesterol ≥ 130 mg/dL, or use of lipid-lowering medication), and coronary artery disease (history of myocardial infarction, coronary artery bypass grafting, or percutaneous coronary intervention).

Neurological deficit at admission was assessed using the National Institutes of Health Stroke Scale (NIHSS) [11]. Recurrent stroke was defined as a new or worsening neurological deficit of vascular origin with neuroimaging-confirmed infarction occurring ≥24 h after the index stroke [12]. Information on reperfusion therapy, including intravenous and intra-arterial thrombolysis, and pre-stroke antiplatelet therapy (aspirin, clopidogrel, cilostazol, triflusal, or combination therapy) was collected.

Stroke subtypes were classified into five etiological categories according to the Trial of Org 10172 in Acute Stroke Treatment (TOAST) criteria [8]: LAD, CE, SVO, stroke of other determined etiology (OD), and stroke of undetermined etiology (UD). Classification was based on clinical presentation together with vascular and cardiac investigations. Two neurologists independently reviewed each case, and disagreements were resolved by consensus. Final subtype assignment was confirmed at the monthly stroke registry meeting.

For laboratory testing, blood samples were obtained from all participants after an overnight fast on the morning following admission. Laboratory data included white blood cell count (×10^3^/μL), platelet count (×10^3^/μL), estimated glomerular filtration rate (eGFR; mL/min/1.73 m^2^), and high-sensitivity CRP. Anemia was defined as a hemoglobin level < 12 g/dL in women and <13 g/dL in men. 

CRP was measured using an immunoturbidimetric assay. During the study period, CRP measurements were performed using the TBA-200FR (Toshiba, Kawasaki, Japan) or cobas c702 (Roche Diagnostics GmbH, Mannheim, Germany) analyzers; detailed assay characteristics and analytical specifications are provided in Table S1.

Cerebral small-vessel disease (CSVD) was assessed on neuroimaging based on the presence of white matter hyperintensities (WMHs) and cerebral microbleeds (CMBs). Periventricular WMHs (PVH) and deep WMHs (DWMH) were graded on FLAIR MRI using the Fazekas scale [13], with a grade > 1 considered indicative of significant WMHs in the respective region [14]. CMBs were defined as small, round or ovoid, homogeneous hypointense lesions on T2*-weighted or susceptibility-weighted MRI sequences [15]. 

### 2.3. Functional Outcomes

Functional outcomes were assessed using the modified Rankin Scale (mRS), a 7-point scale ranging from 0 (no symptoms) to 6 (death), with a score of 5 indicating severe disability [16]. The mRS score was assessed at 3 months (90 days) after stroke onset. The prespecified primary analysis classified an excellent outcome as favorable (mRS 0–1) and mRS 2–6 as unfavorable [16]. Because mRS 0–2 is also commonly used to define functional independence, a sensitivity analysis classified mRS 0–2 as favorable and mRS 3–6 as unfavorable [17].

### 2.4. Statistical Analysis

Normality of continuous variables was assessed using the Kolmogorov–Smirnov test. Because all continuous variables were non-normally distributed, they were summarized as medians with interquartile ranges and compared using the Mann–Whitney *U* test. Categorical variables were summarized as frequencies (percentages) and compared using the Pearson chi-square test. For categorical variables with more than two groups, including TOAST subtypes, post hoc one-versus-rest chi-square analyses were performed when the overall comparison was significant, with Bonferroni correction for multiple comparisons.

Multivariable logistic regression analysis was performed to identify independent predictors of an unfavorable outcome in the overall cohort. Results are reported as adjusted odds ratios (aORs) with 95% confidence intervals (CIs). Separate multivariable models were fitted within each TOAST subtype. The primary analyses retained the prespecified dichotomization of initial stroke severity at NIHSS ≥ 6 [18,19]. In sensitivity analyses, raw NIHSS was modeled as a continuous variable and nonlinearity was assessed using a natural cubic spline. Elevated CRP was prespecified as ≥3 mg/L, a clinically recognized threshold for cardiovascular inflammatory risk [20,21]. Because CRP was markedly right-skewed, additional sensitivity analyses modeled CRP as ln-transformed continuous exposure and evaluated alternative CRP thresholds (≥1 and ≥5 mg/L in addition to the prespecified ≥3 mg/L threshold). SVO refers to the TOAST etiologic subtype of the index stroke, whereas WMHs and CMBs were analyzed as separate imaging markers of chronic CSVD and were not treated as interchangeable constructs. 

Multiplicative CRP×TOAST interaction terms were tested in the overall cohort to formally assess heterogeneity by stroke etiology. For the primary analysis, favorable outcome was defined as mRS 0–1 [16]; an additional sensitivity analysis used mRS 0–2 [17]. The primary analysis used complete cases for model variables. All statistical analyses were performed using R version 4.4.3, and two-sided *p* values < 0.05 were considered statistically significant.

## 3. Results

### 3.1. Baseline Characteristics

Of 3201 patients in the source dataset, 2975 were included in the complete-case primary analysis and 226 (7.1%) were excluded because of missing analysis variables. Missingness was most frequent for CRP (*n* = 162; 5.1%), dyslipidemia (*n* = 95; 3.0%), and NIHSS (*n* = 20; 0.6%). Among the 2975 included patients, 1511 (50.8%) achieved a favorable outcome, whereas 1464 (49.2%) had an unfavorable outcome at 3 months. Compared with patients included in the complete-case analysis, excluded patients showed nominal differences in several clinical and laboratory characteristics, including admission NIHSS, platelet count, eGFR, CRP, recurrent stroke, thrombolysis therapy, and 3-month functional outcome (Table S2). Baseline clinical and laboratory characteristics of the included cohort are summarized in Table 1. 

The overall distribution of TOAST subtypes differed significantly between outcome groups (*p* = 0.005). Post hoc one-versus-rest analyses with Bonferroni correction showed that the CE subtype had a significantly higher proportion of unfavorable outcomes than the rest of the cohort (adjusted *p* = 0.039), whereas no significant differences were observed for the other TOAST subtypes (all adjusted *p* > 0.05).

Patients with unfavorable outcomes also had higher frequencies of recurrent stroke, hypertension, diabetes mellitus, dyslipidemia, and anemia. Elevated CRP was nearly twice as common in the unfavorable outcome group as in the favorable outcome group (15.2% vs. 7.9%, *p* < 0.001).

### 3.2. Independent Predictors in the Overall Cohort

Multivariable logistic regression analysis was performed to identify independent predictors of an unfavorable outcome (Table 2). After adjustment for all covariates, older age, severe initial NIHSS, recurrent stroke, diabetes mellitus, and elevated CRP remained independently associated with an unfavorable outcome (all *p* < 0.05).

**Figure 2:**
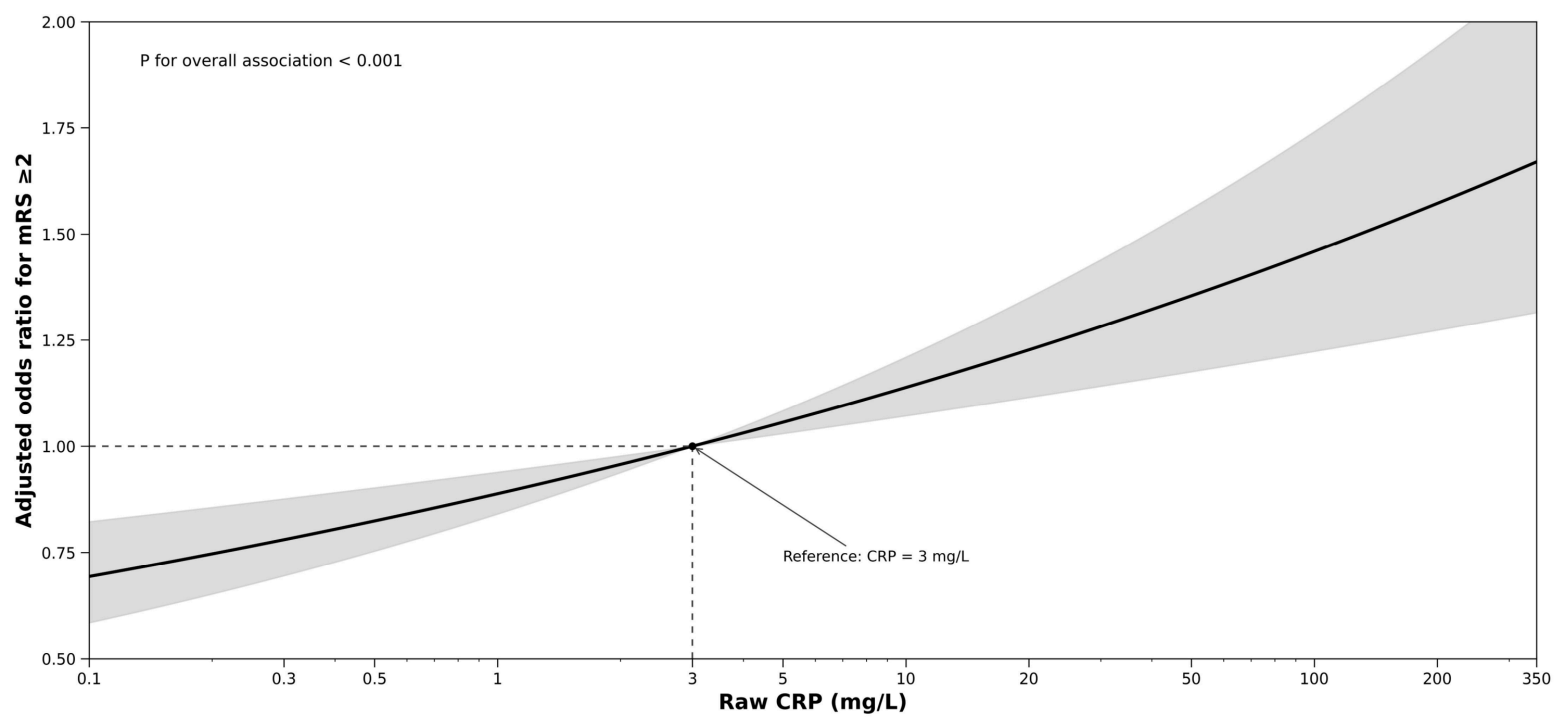
Adjusted association between C-reactive protein level and unfavorable functional outcome at 3 months. The solid line represents the adjusted odds ratio for an unfavorable functional outcome (mRS 2–6) according to CRP level, with CRP 3 mg/L as the reference (OR = 1.0). The shaded area represents the 95% confidence interval. CRP was natural log-transformed and entered as a continuous variable in the multivariable logistic regression model. The model was adjusted for all covariates included in the multivariable logistic regression model presented in Table 2. CRP, C-reactive protein; mRS, modified Rankin Scale; OR, odds ratio; CI, confidence interval.

When CRP was modeled as a log-transformed continuous exposure, the adjusted odds of unfavorable functional outcome (mRS 2–6 at 3 months) generally increased with increasing CRP levels (Figure 2).

### 3.3. Stroke Subtype and Elevated CRP

In the prespecified subtype-stratified analysis using CRP ≥ 3 mg/L and dichotomized NIHSS (Table 3), elevated CRP was associated with an unfavorable 3-month outcome in SVO (aOR, 2.620; 95% CI, 1.094–6.275; *p* = 0.031), but not in LAD or CE. Among 872 patients with SVO, 38 had CRP ≥ 3 mg/L, of whom 30 (78.9%) had an unfavorable outcome (mRS 2–6). 

However, formal CRP×TOAST interaction testing across all five TOAST categories did not demonstrate significant heterogeneity (P_interaction_ = 0.154; Table 4). With nonlinear spline adjustment for raw NIHSS, the association in SVO was attenuated and no longer statistically significant (aOR, 2.243; 95% CI, 0.899–5.598; *p* = 0.083; Table S3), while the five-category interaction remained nonsignificant (P_interaction_ = 0.317; Table 4). 

When CRP was modeled as a continuous log-transformed variable, higher CRP was associated with an unfavorable outcome in the overall cohort across alternative approaches to NIHSS adjustment. The association remained significant after nonlinear adjustment for raw NIHSS using a natural cubic spline (aOR, 1.085 per 1-unit increase in ln[CRP]; 95% CI, 1.030–1.143; *p* = 0.002) (Table S4). In a sensitivity analysis using mRS 0–2 to define a favorable outcome, CRP ≥ 3 mg/L remained associated with an unfavorable outcome (mRS 3–6) in the overall cohort after nonlinear NIHSS adjustment (aOR, 1.388; 95% CI, 1.058–1.819; *p* = 0.018) (Table S3). In contrast, the corresponding association in SVO did not reach statistical significance after nonlinear NIHSS adjustment (aOR, 2.034; 95% CI, 0.882–4.691; *p* = 0.096) (Table S3). Analyses using alternative CRP thresholds of 1 and 5 mg/L are provided in Table 5.

## 4. Discussion

In this cohort of patients with acute ischemic stroke, elevated CRP was associated with unfavorable 3-month functional outcome in the prespecified overall model. In subtype-stratified analyses, CRP ≥ 3 mg/L was associated with unfavorable outcome in SVO, but formal interaction testing across all TOAST categories did not demonstrate significant heterogeneity. Moreover, the SVO estimate was attenuated and no longer statistically significant after flexible nonlinear adjustment for raw NIHSS. These findings therefore do not establish a subtype-specific CRP effect and the observed SVO pattern should be considered hypothesis-generating.

In addition to elevated CRP, older age, diabetes mellitus, severe initial neurological deficit, and recurrent stroke were independently associated with unfavorable outcome. These findings are consistent with previous studies identifying each of these factors as independent predictors of poor functional outcome after ischemic stroke [22,23,24]. Older age likely contributes to poorer recovery through reduced neuroplasticity and diminished collateral reserve [25,26]. Diabetes impairs post-stroke recovery by promoting endothelial dysfunction, chronic inflammation, impaired neurovascular repair, and reduced neuroplasticity [27,28]. A severe initial neurological deficit probably reflects greater infarct volume and involvement of eloquent brain regions and remains the strongest predictor of functional outcome across stroke subtypes [23,24]. Recurrent stroke further increases the risk of long-term disability following the index event [22,23,24].

Elevated CRP was associated with unfavorable outcome in the prespecified overall model, consistent with previous clinical studies and meta-analyses [4,5,6,7]. CRP may reflect systemic inflammatory burden as well as stroke severity and atherosclerotic disease [28,29,30,31,32]. Because CRP was assessed at a single study time point and the exact onset-to-sampling interval was unavailable, the present study cannot distinguish transient acute inflammatory responses from persistent inflammatory activity; serial CRP measurements should be evaluated prospectively.

The prespecified subgroup analysis suggested an association between CRP ≥3 mg/L and unfavorable outcome in SVO. Prior literature has linked inflammation with endothelial dysfunction and blood–brain barrier vulnerability in cerebral small-vessel disease [33,34] and has reported prognostic associations of CRP in lacunar or small subcortical infarction [35,36]. However, TOAST SVO is an etiologic classification of the index stroke and should not be equated with chronic CSVD imaging burden. In the present study, the available WMH and CMB markers did not provide evidence for a causal pathway linking CRP, chronic small-vessel pathology, and outcome. Proposed mechanisms involving endothelial dysfunction, blood–brain barrier injury, oxidative stress, or microvascular vulnerability therefore remain speculative in the context of this observational analysis. The small number of CRP-elevated SVO patients (*n* = 38) and the wide confidence interval further limit the precision of this subgroup estimate.

LAD is characterized by atherosclerotic plaque disease, and CRP has been studied as a marker of atherosclerotic inflammation [37]. Previous studies have reported prognostic associations of CRP in LAD [4,29]. In the present study, however, CRP ≥ 3 mg/L was not significantly associated with outcome in LAD in the prespecified model. This null subgroup result should not be interpreted as evidence that the effect differs from other TOAST categories because the formal all-category interaction was nonsignificant. Detailed plaque characteristics, stenosis severity, infarct volume, and collateral status were not systematically available and may contribute to residual confounding [38,39,40].

CE had a high proportion of unfavorable outcomes in our cohort. Diabetes showed an inverse association with unfavorable outcomes in the CE model, but this unexpected finding should not be interpreted as a protective effect and may reflect residual confounding or subgroup instability. The present dataset does not contain sufficient treatment-detail data to determine whether anticoagulation or closer cardiovascular management explains this association. Further studies are needed to clarify this finding.

Elevated CRP was not independently associated with functional outcome in CE in the prespecified subgroup model. Outcomes after cardioembolic stroke may be strongly influenced by infarct burden, large-vessel occlusion, and cardiac function [41,42,43,44], but these mechanisms were not directly tested here. In addition, non-stroke inflammatory or cardiac conditions can influence CRP. Because detailed infarct volume, large-vessel occlusion, cardiac inflammatory conditions, and other potential causes of CRP elevation were not systematically available, mechanistic interpretation of the CE result should remain cautious.

A major strength of this study is the large registry cohort with TOAST subtype-stratified analyses and the availability of raw NIHSS, mRS, and CRP for sensitivity analyses. Nevertheless, several limitations should be emphasized. First, exact stroke-onset, admission, and CRP-sampling times were not available in the registry extract; therefore, the onset-to-CRP interval could not be quantified or adjusted for despite the dynamic behavior of CRP after ischemic stroke. Second, the primary analysis was based on complete cases, with 226 of 3201 patients (7.1%) excluded because of missing analysis variables. Exploratory comparisons suggested that excluded patients differed from the analyzed cohort in several measured characteristics; therefore, the possibility of selection bias due to complete-case analysis should be considered. Third, the prespecified NIHSS dichotomization may have left residual confounding by stroke severity; importantly, the SVO association was attenuated after nonlinear adjustment for raw NIHSS. Fourth, detailed information on infarct volume and location, large-vessel occlusion, stenosis severity, collateral status, mechanical thrombectomy, and post-stroke rehabilitation was not systematically available; therefore, residual confounding related to stroke burden, vascular anatomy, acute treatment, and rehabilitation intensity cannot be excluded. Fifth, potential non-stroke causes of CRP elevation, including malignancy, autoimmune disease, and intercurrent inflammatory or infectious conditions, were not systematically available. Sixth, CRP was assessed at a single study time point, precluding evaluation of persistent inflammatory activity. In addition, two analyzer systems were used during the earlier study period. Although both used an immunoturbidimetric assay, differences between the analyzer and reagent systems may have introduced some measurement variability. Seventh, the relatively small number of patients with elevated CRP in SVO and the small OD subgroup produced imprecise estimates. Finally, the registry-based observational design precludes causal inference and may limit generalizability.

In conclusion, elevated CRP was associated with an unfavorable 3-month functional outcome in the prespecified overall analysis, with a higher point estimate observed in the SVO subgroup. However, formal interaction testing did not demonstrate significant heterogeneity across all TOAST subtypes, and the SVO estimate was attenuated in sensitivity analyses using nonlinear adjustment for raw NIHSS. These findings support further investigation of CRP as a prognostic biomarker after ischemic stroke but do not establish a subtype-specific association or justify screening or treatment decisions based on CRP alone.

## Figures and Tables

**Table 1:** Clinical and laboratory characteristics by functional outcome.

Characteristics	Total (*n* = 2975)	Favorable Outcome (*n* = 1511)	Unfavorable Outcome (*n* = 1464)	*p* Value
*Demographics*				
Age (years)	69.0 [60.0–79.0]	66.0 [59.0–77.0]	72.0 [62.0–81.0]	<0.001
Sex (male)	1834 (61.6%)	978 (64.7%)	856 (58.5%)	<0.001
*Comorbidities*				
Hypertension	2037 (68.5%)	995 (65.9%)	1042 (71.2%)	0.002
Diabetes mellitus	1417 (47.6%)	653 (43.2%)	764 (52.2%)	<0.001
Dyslipidemia	1574 (52.9%)	837 (55.4%)	737 (50.3%)	0.006
Coronary artery disease	276 (9.3%)	121 (8.0%)	155 (10.6%)	0.018
*Clinical and stroke profiles*				
Severe initial NIHSS score	909 (30.6%)	285 (18.9%)	624 (42.6%)	<0.001
Recurrent stroke	581 (19.5%)	234 (15.5%)	347 (23.7%)	<0.001
Thrombolysis therapy	336 (11.3%)	137 (9.1%)	199 (13.6%)	<0.001
Pre-stroke antiplatelet therapy	857 (28.8%)	398 (26.3%)	459 (31.4%)	0.003
*TOAST classification*				0.005
LAD	669 (22.5%)	324 (21.4%)	345 (23.6%)	(0.897)
CE	618 (20.8%)	284 (18.8%)	334 (22.8%)	(0.039)
SVO	872 (29.3%)	469 (31.0%)	403 (27.5%)	(0.195)
OD	113 (3.8%)	68 (4.5%)	45 (3.1%)	(0.262)
UD	703 (23.6%)	366 (24.2%)	337 (23.0%)	(1.000)
*Laboratory findings*				
White blood cell (103/μL)	7.6 [6.2–9.4]	7.4 [6.1–9.2]	7.8 [6.3–9.7]	<0.001
Anemia	701 (23.6%)	307 (20.3%)	394 (26.9%)	<0.001
Platelet (103/μL)	223 [185–266]	224 [187–267]	222 [184–266]	0.579
eGFR (mL/min/1.73 m^2^)	83.8 [65.0–100.0]	86.0 [69.4–102.0]	80.6 [61.3–97.5]	<0.001
Elevated CRP	342 (11.5%)	120 (7.9%)	222 (15.2%)	<0.001
*Neuroimaging markers of CSVD*				
PVH	1042 (35.0%)	480 (31.8%)	562 (38.4%)	<0.001
DWMH	933 (31.4%)	433 (28.7%)	500 (34.2%)	0.001
CMBs	608 (20.4%)	282 (18.7%)	326 (22.3%)	0.017

Data are presented as median [interquartile range] or n (%). *p* values were calculated using the Mann–Whitney *U* test for continuous variables and the chi-square test for categorical variables. *p* values in parentheses represent post hoc one-versus-rest comparisons against all other subgroups, adjusted by the Bonferroni method for multiple comparisons. NIHSS, National Institutes of Health Stroke Scale; TOAST, Trial of Org 10172 in Acute Stroke Treatment; LAD, large-artery disease; CE, cardioembolism; SVO, small-vessel occlusion; OD, other determined etiology; UD, undetermined etiology; eGFR, estimated glomerular filtration rate; CSVD, cerebral small-vessel disease; PVH, periventricular white matter hyperintensities; DWMH, deep white matter hyperintensities; CMBs, cerebral microbleeds.

**Table 2:** Logistic regression analysis of unfavorable outcome in the overall cohort.

Characteristics	Crude OR [95% CI]	Adjusted OR [95% CI]
Demographics		
Age (years)	**1.029 [1.022–1.036]**	**1.016 [1.008–1.024]**
Sex (male)	**0.767 [0.662–0.890]**	0.942 [0.798–1.112]
Comorbidities		
Hypertension	**1.281 [1.096–1.496]**	1.083 [0.913–1.284]
Diabetes mellitus	**1.434 [1.241–1.657]**	**1.352 [1.158–1.578]**
Dyslipidemia	**0.816 [0.707–0.943]**	0.979 [0.835–1.148]
Coronary artery disease	**1.360 [1.060–1.746]**	1.116 [0.851–1.464]
Clinical and stroke profiles		
Severe initial NIHSS score	**3.196 [2.709–3.770]**	**2.807 [2.328–3.385]**
Recurrent stroke	**1.695 [1.410–2.038]**	**1.445 [1.174–1.778]**
Thrombolysis therapy	**1.578 [1.253–1.987]**	0.996 [0.769–1.292]
Pre-stroke antiplatelet therapy	**1.277 [1.089–1.497]**	0.938 [0.777–1.132]
Laboratory findings		
White blood cell	**1.027 [1.006–1.049]**	0.998 [0.979–1.017]
Anemia	**1.444 [1.218–1.712]**	1.075 [0.887–1.303]
Platelet	1.000 [0.999–1.001]	1.001 [1.000–1.002]
eGFR	**0.994 [0.992–0.997]**	0.999 [0.996–1.002]
Elevated CRP	**2.072 [1.638–2.621]**	**1.301 [1.003–1.687]**
Neuroimaging markers of CSVD		
PVH	**1.338 [1.151–1.557]**	0.921 [0.747–1.137]
DWMH	**1.291 [1.106–1.508]**	0.967 [0.792–1.203]
CMBs	**1.248 [1.044–1.493]**	1.119 [0.910–1.376]

Crude ORs were estimated using univariable logistic regression. Adjusted ORs were estimated from a multivariable logistic regression model including all variables listed in the table. Values in bold font indicate statistical significance (*p* < 0.05). OR, odds ratio; CI, confidence interval; NIHSS, National Institutes of Health Stroke Scale; eGFR, estimated glomerular filtration rate; CSVD, cerebral small-vessel disease; PVH, periventricular white matter hyperintensities; DWMH, deep white matter hyperintensities; CMBs, cerebral microbleeds.

**Table 3:** Multivariable logistic regression analysis of an unfavorable outcome stratified by TOAST subtype.

Characteristics	LAD (*n* = 669)	CE (*n* = 618)	SVO (*n* = 872)	OD (*n* = 113)	UD (*n* = 703)
*Demographics*					
Age (years)	1.012 [0.994–1.031]	1.010 [0.991–1.029]	**1.036 [1.020–1.053]**	**0.927 [0.871–0.986]**	1.013 [0.996–1.030]
Sex (male)	1.003 [0.685–1.469]	0.708 [0.488–1.027]	0.825 [0.597–1.138]	0.578 [0.197–1.701]	1.223 [0.855–1.749]
*Comorbidities*					
Hypertension	0.852 [0.592–1.226]	1.256 [0.845–1.867]	1.030 [0.749–1.416]	1.505 [0.451–5.015]	1.338 [0.918–1.951]
Diabetes mellitus	1.358 [0.971–1.900]	**0.568 [0.394–0.818]**	**1.500 [1.117–2.015]**	2.245 [0.686–7.339]	**2.092 [1.511–2.896]**
Dyslipidemia	1.049 [0.746–1.476]	1.175 [0.821–1.683]	0.806 [0.592–1.097]	3.689 [0.993–13.705]	0.966 [0.688–1.357]
Coronary artery disease	1.245 [0.655–2.368]	1.076 [0.662–1.749]	1.254 [0.636–2.473]	2.580 [0.123–54.190]	0.989 [0.593–1.649]
*Clinical and stroke profiles*					
Severe initial NIHSS score	**3.199 [2.185–4.683]**	**2.881 [1.971–4.210]**	**4.187 [2.460–7.126]**	**3.547 [1.088–11.567]**	**3.195 [2.169–4.708]**
Recurrent stroke	1.486 [0.918–2.404]	1.522 [0.997–2.324]	1.231 [0.776–1.950]	1.626 [0.351–7.536]	1.460 [0.971–2.195]
Thrombolysis therapy	1.027 [0.577–1.828]	1.426 [0.900–2.258]	0.764 [0.344–1.697]	2.297 [0.196–26.957]	0.772 [0.460–1.297]
Pre-stroke antiplatelet therapy	1.009 [0.660–1.544]	0.704 [0.480–1.031]	1.181 [0.793–1.761]	**5.689 [1.179–27.439]**	0.819 [0.556–1.205]
*Laboratory findings*					
White blood cell	0.977 [0.928–1.028]	0.994 [0.954–1.036]	**1.081 [1.010–1.157]**	1.008 [0.950–1.070]	0.990 [0.936–1.047]
Anemia	1.137 [0.750–1.725]	1.340 [0.883–2.034]	1.064 [0.706–1.603]	0.986 [0.320–3.035]	1.150 [0.783–1.691]
Platelet	1.000 [0.998–1.003]	1.002 [0.999–1.004]	1.000 [0.998–1.002]	1.001 [0.996–1.006]	1.000 [0.998–1.002]
eGFR	1.001 [0.995–1.007]	0.998 [0.992–1.005]	0.998 [0.992–1.004]	0.983 [0.965–1.001]	1.001 [0.996–1.006]
Elevated CRP	1.562 [0.905–2.696]	1.206 [0.731–1.988]	**2.620 [1.094–6.275]**	**7.249 [1.381–38.051]**	1.238 [0.748–2.047]
*Neuroimaging of CSVD*					
PVH	0.999 [0.638–1.562]	1.013 [0.617–1.665]	1.026 [0.703–1.496]	1.356 [0.212–8.671]	0.758 [0.481–1.192]
DWMH	1.044 [0.668–1.634]	0.898 [0.551–1.464]	1.005 [0.688–1.469]	3.397 [0.471–24.479]	0.888 [0.567–1.390]
CMBs	1.515 [0.911–2.519]	1.167 [0.712–1.911]	0.984 [0.676–1.432]	1.549 [0.350–6.858]	1.159 [0.769–1.748]

Data are expressed as odds ratios [95% confidence intervals], which were estimated from separate multivariable logistic regression models including all variables listed in the table. Values in bold font indicate statistical significance (*p* < 0.05). LAD, large-artery disease; CE, cardioembolism; SVO, small-vessel occlusion; OD, other determined etiology; UD, undetermined etiology; NIHSS, National Institutes of Health Stroke Scale; eGFR, estimated glomerular filtration rate; CSVD, cerebral small-vessel disease; PVH, periventricular white matter hyperintensities; DWMH, deep white matter hyperintensities; CMBs, cerebral microbleeds.

**Table 4:** Likelihood-ratio tests for the interaction between elevated C-reactive protein and TOAST subtypes on functional outcomes.

Outcome	Comparison	NIHSS	LR	df	P_interaction_
mRS 0–1 favorable	All 5 TOAST	NIHSS ≥ 6	6.684	4	0.154
		NIHSS continuous	7.344	4	0.119
		NIHSS spline	4.726	4	0.317
	LAD/CE/SVO	NIHSS ≥ 6	6.092	2	0.048
		NIHSS continuous	6.651	2	0.036
		NIHSS spline	4.319	2	0.115
mRS 0–2 favorable	All 5 TOAST	NIHSS ≥ 6	6.463	4	0.167
		NIHSS continuous	7.600	4	0.107
		NIHSS spline	4.537	4	0.338
	LAD/CE/SVO	NIHSS ≥ 6	5.613	2	0.060
		NIHSS continuous	6.149	2	0.046
		NIHSS spline	4.006	2	0.135

P_interaction_ values were derived from likelihood-ratio tests comparing multivariable logistic regression models with and without multiplicative CRP×TOAST interaction terms. The interaction tests were conducted across all five TOAST etiologic categories and separately restricted to the three major stroke subtypes (LAD, CE, and SVO). NIHSS spline indicates that the continuous NIHSS score was flexibly modeled using a natural cubic spline with 4 degrees of freedom to adjust for potential nonlinear effects. NIHSS, National Institutes of Health Stroke Scale; LR, likelihood ratio; df, degrees of freedom; mRS, modified Rankin Scale; TOAST, Trial of Org 10172 in Acute Stroke Treatment; LAD, large-artery disease; CE, cardioembolism; SVO, small-vessel occlusion.

**Table 5:** Sensitivity analyses evaluating alternative C-reactive protein thresholds for predicting unfavorable functional outcomes across TOAST subtypes according to three approaches to NIHSS adjustment.

CRP Threshold	TOAST (*n*)	Elevated CRP (*n)*	NIHSS ≥ 6 Adjustment aOR (95% CI)	Continuous NIHSS Adjustment aOR (95% CI)	NIHSS Spline Adjustment aOR (95% CI)
1 mg/L	Overall (2975)	608	**1.309 (1.064–1.610)**	**1.291 (1.049–1.589)**	1.178 (0.950–1.462)
	LAD (669)	151	1.002 (0.655–1.531)	1.002 (0.656–1.531)	0.897 (0.577–1.394)
	CE (618)	171	**1.537 (1.006–2.347)**	**1.556 (1.021–2.372)**	1.463 (0.944–2.268)
	SVO (872)	81	**1.940 (1.121–3.357)**	1.662 (0.934–2.957)	1.765 (0.987–3.155)
3 mg/L	Overall (2975)	342	**1.301 (1.003–1.687)**	1.279 (0.985–1.661)	1.212 (0.925–1.587)
	LAD (669)	87	1.562 (0.905–2.696)	1.640 (0.951–2.829)	1.423 (0.815–2.486)
	CE (618)	98	1.206 (0.731–1.988)	1.223 (0.744–2.009)	1.201 (0.715–2.017)
	SVO (872)	38	**2.620 (1.094–6.275)**	2.005 (0.806–4.988)	2.243 (0.899–5.598)
5 mg/L	Overall (2975)	200	0.819 (0.595–1.128)	0.786 (0.568–1.087)	0.765 (0.550–1.065)
	LAD (669)	51	1.018 (0.529–1.959)	1.112 (0.579–2.136)	0.870 (0.449–1.684)
	CE (618)	59	0.669 (0.366–1.222)	0.629 (0.343–1.153)	0.655 (0.349–1.229)
	SVO (872)	17	0.999 (0.316–3.157)	0.854 (0.251–2.899)	1.084 (0.310–3.789)

Elevated CRP was defined separately at thresholds of ≥1, ≥3, and ≥5 mg/L. The outcome was unfavorable functional status at 3 months (mRS 2–6). Adjusted odds ratios were estimated using multivariable logistic regression. The three NIHSS specifications were (1) dichotomized admission NIHSS (≥6 vs. <6), (2) raw admission NIHSS modeled as a continuous covariate, and (3) raw admission NIHSS modeled using a natural cubic spline with 4 degrees of freedom. All models were additionally adjusted for age, sex, hypertension, diabetes mellitus, dyslipidemia, coronary artery disease, recurrent stroke, thrombolysis therapy, pre-stroke antiplatelet therapy, white blood cell count, anemia, platelet count, eGFR, periventricular white matter hyperintensity, deep white matter hyperintensity, and cerebral microbleeds. Values with *p* < 0.05 are shown in bold. CRP, C-reactive protein; NIHSS, National Institutes of Health Stroke Scale; TOAST, Trial of Org 10172 in Acute Stroke Treatment; LAD, large-artery disease; CE, cardioembolism; SVO, small-vessel occlusion; aOR, adjusted odds ratio; CI, confidence interval; mRS, modified Rankin Scale; eGFR, estimated glomerular filtration rate.

## Data Availability

The raw data supporting the conclusions of this article will be made available by the authors upon request.

## Supplementary Materials

The following supporting information can be downloaded at: https://www.mdpi.com/article/10.3390/jcm15187192/s1. Table S1. C-reactive protein assay characteristics and analytical specifications during the study period. Table S2. Comparison of baseline characteristics between patients included in the complete-case analysis and those excluded because of missing analysis variables. Table S3. Sensitivity analyses for the association between elevated C-reactive protein (≥3 mg/L) and unfavorable functional outcomes using alternative NIHSS adjustments and mRS cutoffs by TOAST subtype. Table S4. Sensitivity analyses evaluating continuous log-transformed C-reactive protein for predicting functional outcomes by TOAST subtype under varying NIHSS adjustment models.
