# Supplementary material for: Association of C-Reactive Protein with Functional Outcome After Acute Ischemic Stroke Across TOAST Subtypes
Source: J Clin Med. 2026 Sep 16;15(18):7192. doi: 10.3390/jcm15187192 (PMC13607515; doi:10.3390/jcm15187192)
Supplement: Supplementary file 1 [file jcm-15-07192-s001.zip › jcm-4525936-supplementary.pdf]

Supplementary Materials

Table S1. C-reactive protein assay characteristics and analytical specifications during the study period.

**Period of use**	March 2013-May 2016	March 2013-May 2021
**Analyzer**	TBA-200FR	Cobas c702
**Manufacturer**	Toshiba, Japan	Roche Diagnostics GmbH, Germany
**Reagent**	Denka Seiken, Japan	Roche Diagnostics GmbH, Germany
**Analytical range**	0.1–320.0 mg/L	0.3–350.0 mg/L
**Detection limit**	0.1 mg/L	0.3 mg/L
**Assay method**	Immunoturbidimetric assay	Immunoturbidimetric assay

During March 2013–May 2016, CRP testing was performed using either of two analyzer/reagent systems: TBA-200FR with Denka Seiken reagent or Cobas c702 with Roche Diagnostics reagent. From June 2016 through the end of the study period (May 2021), the Cobas c702 system with Roche Diagnostics reagent was used.

Table S2. Comparison of baseline characteristics between patients included in the complete-case analysis and those excluded because of missing analysis variables.

Characteristic	Type	Included, n	Available n in excluded group	Included in complete-case analysis (n=2,975)	Excluded because of missing analysis variables (n=226)	*P* value
Age (years)	Continuous	2975	226	69 [60–79]	69 [61–79]	0.514
Sex (male)	Categorical	2975	226	1834 (61.6%)	147 (65.0%)	0.346
Hypertension	Categorical	2975	226	2037 (68.5%)	147 (65.0%)	0.321
Diabetes mellitus	Categorical	2975	226	1417 (47.6%)	88 (38.9%)	**0.012**
Dyslipidemia	Categorical	2975	131	1574 (52.9%)	60 (45.8%)	0.132
Coronary artery disease	Categorical	2975	226	276 (9.3%)	25 (11.1%)	0.443
Initial NIHSS score	Continuous	2975	206	3 [1–7]	2 [1–6]	**0.042**
Recurrent stroke	Categorical	2975	226	581 (19.5%)	24 (10.6%)	**0.001**
Thrombolysis therapy	Categorical	2975	226	336 (11.3%)	13 (5.8%)	**0.014**
Pre-stroke antiplatelet therapy	Categorical	2975	226	857 (28.8%)	71 (31.4%)	0.449
TOAST subtype	Categorical	2975	226	LAD 22.5%; CE 20.8%; SVO 29.3%; OD 3.8%; UD 23.6%	LAD 22.6%; CE 18.6%; SVO 28.3%; OD 4.4%; UD 26.1%	0.859
White blood cell count (×10^3/μL)	Continuous	2975	222	7.59 [6.19–9.41]	7.46 [5.87–9.21]	0.230
Anemia	Categorical	2975	222	701 (23.6%)	94 (42.3%)	**<0.001**
Platelet count (×10^3/μL)	Continuous	2975	226	223 [185–266]	210 [161–261]	**0.004**
eGFR (mL/min/1.73 m^2)	Continuous	2975	224	83.8 [65.0–100.0]	78.9 [55.6–100.1]	**0.024**
CRP (mg/L)	Continuous	2975	64	0.180 [0.067–0.700]	0.846 [0.154–2.591]	**<0.001**
Periventricular WMH	Categorical	2975	226	1042 (35.0%)	68 (30.1%)	0.152
Deep WMH	Categorical	2975	226	933 (31.4%)	62 (27.4%)	0.248
Cerebral microbleeds	Categorical	2975	226	608 (20.4%)	46 (20.4%)	1.000
Unfavorable 3-month outcome (mRS 2–6)	Categorical	2975	226	1464 (49.2%)	65 (28.8%)	**<0.001**

Data are presented as median [interquartile range] for continuous variables and n (%) for categorical variables. Because patients were excluded for missing data in one or more analysis variables, the number of available observations in the excluded group varied across variables; the corresponding nonmissing denominator is shown for each variable.

P values were calculated using the Mann–Whitney U test for continuous variables and the Pearson chi-square test for categorical variables. Continuity correction was applied to binary categorical comparisons. Values in bold font indicate statistical significance ($P < 0.05$). These comparisons were exploratory and descriptive, and no adjustment for multiple comparisons was applied.

NIHSS, National Institutes of Health Stroke Scale; TOAST, Trial of Org 10172 in Acute Stroke Treatment; LAD, large-artery disease; CE, cardioembolism; SVO, small-vessel occlusion; OD, other determined etiology; UD, undetermined etiology; eGFR, estimated glomerular filtration rate; CRP, C-reactive protein; WMH, white matter hyperintensity; mRS, modified Rankin Scale.

Table S3. Sensitivity analyses for the association between elevated C-reactive protein (≥3 mg/L) and unfavorable functional outcomes using alternative NIHSS adjustments and mRS cutoffs by TOAST subtype.

TOAST	Outcome	NIHSS	OR (95% CI)	Elevated CRP	Elevated CRP with unfavorable outcome
Overall (*n*=2,975)	mRS 0–1 favorable	NIHSS ≥6	**1.301 (1.003–1.687)**	(*n*=342)	(*n*=222)
		NIHSS continuous	1.279 (0.985–1.661)	(*n*=342)	(*n*=222)
		NIHSS spline	1.212 (0.925–1.587)	(*n*=342)	(*n*=222)
	mRS 0–2 favorable	NIHSS ≥6	**1.529 (1.174–1.990)**	(*n*=342)	(*n*=178)
		NIHSS continuous	**1.492 (1.144–1.946)**	(*n*=342)	(*n*=178)
		NIHSS spline	**1.388 (1.058–1.819)**	(*n*=342)	(*n*=178)
LAD (*n*=669)	mRS 0–1 favorable	NIHSS ≥6	1.562 (0.905–2.696)	(*n*=87)	(*n*=60)
		NIHSS continuous	1.640 (0.951–2.829)	(*n*=87)	(*n*=60)
		NIHSS spline	1.423 (0.815–2.486)	(*n*=87)	(*n*=60)
	mRS 0–2 favorable	NIHSS ≥6	1.628 (0.958–2.767)	(*n*=87)	(*n*=49)
		NIHSS continuous	**1.732 (1.019–2.944)**	(*n*=87)	(*n*=49)
		NIHSS spline	1.522 (0.888–2.609)	(*n*=87)	(*n*=49)
CE (*n*=618)	mRS 0–1 favorable	NIHSS ≥6	1.206 (0.731–1.988)	(*n*=98)	(*n*=59)
		NIHSS continuous	1.223 (0.744–2.009)	(*n*=98)	(*n*=59)
		NIHSS spline	1.201 (0.715–2.017)	(*n*=98)	(*n*=59)
	mRS 0–2 favorable	NIHSS ≥6	1.537 (0.905–2.61)	(*n*=98)	(*n*=47)
		NIHSS continuous	1.475 (0.874–2.487)	(*n*=98)	(*n*=47)
		NIHSS spline	1.493 (0.864–2.579)	(*n*=98)	(*n*=47)
SVO (*n*=872)	mRS 0–1 favorable	NIHSS ≥6	**2.620 (1.094–6.275)**	(*n*=38)	(*n*=30)
		NIHSS continuous	2.005 (0.806–4.988)	(*n*=38)	(*n*=30)
		NIHSS spline	2.243 (0.899–5.598)	(*n*=38)	(*n*=30)
	mRS 0–2 favorable	NIHSS ≥6	**2.566 (1.163–5.665)**	(*n*=38)	(*n*=23)
		NIHSS continuous	1.922 (0.846–4.366)	(*n*=38)	(*n*=23)
		NIHSS spline	2.034 (0.882–4.691)	(*n*=38)	(*n*=23)
OD (*n*=113)	mRS 0–1 favorable	NIHSS ≥6	**7.249 (1.381–38.053)**	(*n*=21)	(*n*=12)
		NIHSS continuous	**6.201 (1.168–32.933)**	(*n*=21)	(*n*=12)
		NIHSS spline	**44.362 (4.537–433.724)**	(*n*=21)	(*n*=12)
	mRS 0–2 favorable	NIHSS ≥6	**7.748 (1.445–41.552)**	(*n*=21)	(*n*=10)
		NIHSS continuous	5.699 (0.995–32.637)	(*n*=21)	(*n*=10)
		NIHSS spline	**29.595 (3.311–264.504)**	(*n*=21)	(*n*=10)
UD (*n*=703)	mRS 0–1 favorable	NIHSS ≥6	1.238 (0.748–2.047)	(*n*=98)	(*n*=61)
		NIHSS continuous	1.166 (0.702–1.934)	(*n*=98)	(*n*=61)
		NIHSS spline	1.257 (0.742–2.128)	(*n*=98)	(*n*=61)
	mRS 0–2 favorable	NIHSS ≥6	1.232 (0.738–2.056)	(*n*=98)	(*n*=49)
		NIHSS continuous	1.145 (0.681–1.924)	(*n*=98)	(*n*=49)
		NIHSS spline	1.214 (0.715–2.063)	(*n*=98)	(*n*=49)

Elevated CRP was defined as a CRP level ≥3 mg/L. The "Elevated CRP" column indicates the number of patients with elevated CRP in each group, whereas the "Elevated CRP with unfavorable outcome" column indicates the number of these patients who had an unfavorable functional outcome according to the corresponding mRS cutoff. NIHSS spline indicates that the continuous NIHSS score was flexibly modeled using a natural cubic spline with 4 degrees of freedom (df=4) to adjust for potential nonlinear effects. Values in bold font indicate statistical significance ($P < 0.05$).

NIHSS, National Institutes of Health Stroke Scale; TOAST, Trial of Org 10172 in Acute Stroke Treatment; LAD, large-artery disease; CE, cardioembolism; SVO, small-vessel occlusion; OD, other determined etiology; UD, undetermined etiology; OR, odds ratio; CI, confidence interval; mRS, modified Rankin Scale.

Table S4. Sensitivity analyses evaluating continuous log-transformed C-reactive protein for predicting functional outcomes by TOAST subtype under varying NIHSS adjustment models.

TOAST	NIHSS	OR (95% CI)
Overall	NIHSS ≥6	**1.115 (1.061–1.172)**
(*n*=2,975)	NIHSS continuous	**1.113 (1.059–1.171)**
	NIHSS spline	**1.085 (1.030–1.143)**
LAD	NIHSS ≥6	**1.117 (1.006–1.240)**
(*n*=669)	NIHSS continuous	**1.128 (1.016–1.251)**
	NIHSS spline	1.072 (0.963–1.193)
CE	NIHSS ≥6	**1.202 (1.075–1.345)**
(*n*=618)	NIHSS continuous	**1.207 (1.079–1.349)**
	NIHSS spline	**1.199 (1.068–1.347)**
SVO	NIHSS ≥6	1.120 (0.997–1.257)
(*n*=872)	NIHSS continuous	1.072 (0.950–1.210)
	NIHSS spline	1.085 (0.959–1.228)
OD	NIHSS ≥6	1.302 (0.972–1.745)
(*n*=113)	NIHSS continuous	1.256 (0.929–1.697)
	NIHSS spline	**1.874 (1.252–2.803)**
UD	NIHSS ≥6	1.106 (0.996–1.228)
(*n*=703)	NIHSS continuous	1.103 (0.993–1.225)
	NIHSS spline	1.087 (0.975–1.213)

NIHSS spline indicates that the continuous NIHSS score was flexibly modeled using a natural cubic spline with 4 degrees of freedom to adjust for potential nonlinear effects. Because CRP values were markedly right-skewed, they were transformed using the natural logarithm before analysis. ORs are expressed per 1-unit increase in ln(CRP). Values in bold font indicate statistical significance ($P < 0.05$).

NIHSS, National Institutes of Health Stroke Scale; TOAST, Trial of Org 10172 in Acute Stroke Treatment; LAD, large-artery disease; CE, cardioembolism; SVO, small-vessel occlusion; OD, other determined etiology; UD, undetermined etiology; OR, odds ratio; CI, confidence interval.
